# Buffalo Academy of Medicine

**Published:** 1903-08

**Authors:** F. W. McGuire


Respectfully submitted,

### Buffalo Academy of Medicine

Reported by F. W. McGUIRE, M. D., Secretary.

*Annual Meeting, June 9, 1903.*

The annual meeting of the Buffalo Academy of Medicine was held at the Academy rooms, Public Library Building, Tuesday evening, June 9, 1903.

The president, Dr. Herman E. Hayd, called the meeting to order at 9 o'clock p.m.

The minutes of the last annual meeting were read and approved.

The secretary of the academy and of the council presented the following reports for the Academy year of 1902-1903, which were received and on motion ordered to be spread on the minutes.

ANNUAL REPORT OF THE SECRETARY.

The Academy has been unfortunate, during the year, in losing five of its influential members by death,—namely, Thomas Lothrop, Jacob M. Kraus, Herman Mynter, William C. Fritz, and Jacob F. Meyer. The Academy also has lost eight members, dropped for nonpayment of dues, and two by resignation, a total loss of fifteen members. Eight new names have been added.

While there has been a slight decrease of the membership of the Academy, there has been a marked increase in the attendance at its meetings, which can be accounted for easily by the excellent programs prepared by the officers of the different sections.

The average attendance at the meetings has increased from 44 to 55.

During the summer of 1902, a program was prepared for the entire season, and a copy of it sent to each member of the Academy.

There have been five stated meetings. In September, the section of medicine provided the following program: Syphilis of the nervous system, Floyd S. Crego; Cerebral hemorrhage, following injury to the spine, Sydney A. Dunham. The attendance, 28.

In November the surgical section provided the following program: The physics and therapeutic value of the (Röntgen) cathode, and ultra violet rays, with certain references to the electromagnetic theory of light, and exhibition of new instruments for phototherapy, Roswell Park. Attendance, 108.

In January the section of pathology provided the following: Surgical treatment of puerperal infection, Matthew D. Mann. Attendance, 48.

In March the section of ophthalmology supplied the program as follows: A consideration of the value of topical applications to the upper air tract, Frank Whitehill Hinkel. Attendance, 35.

In May the section of obstetrics and gynecology was the provider, as follows: Should the uterus be removed when operating for double pus tubes? Matthew D. Mann. Prophylaxis in obstetrics, Ludwig Schroeter. Attendance, 21.

A special meeting of the Academy was held May 20, at which Professor C. A. Ewald, of Berlin, gave an address on The early diagnosis of gastric cancer. Attendance, 90.

The Council held six meetings during the year, with an average attendance of 10.66.

ANNUAL REPORT OF THE TREASURER.

*Current Fund.*

| | | |
|---|---|---|
| Balance on hand, June 17, 1902 | $387.37 | |
| Initiation fees seven resident Fellows | 70.00 | |
| Dues received from Fellows | 848.00 | |
| Received for use of telephone | .40 | |
| Interest, Fidelity Trust Company | 15.41 | |
| | | $1,321.18 |

Expenses:

| | | |
|---|---|---|
| Printing, postage and stationery | $210.73 | |
| Janitor's service, including elevator | 57.50 | |
| Rent | 43.00 | |
| Stereopticon | 25.00 | |
| Telephone | 19.10 | |
| Repairs to property | 2.00 | |
| Contribution to Virchow Monument Fund | 25.00 | |
| Collations | 50.00 | |
| Guests' expenses—Medical Section | 72.90 | |
| Guests' expenses—Surgical Section | 46.25 | |
| Guests' expenses—Pathological Section ($62.00 for last year) | 162.00 | |
| Guests' expenses—O., L. and O. Section | 42.00 | |
| Total expenses | $755.48 | |
| Transferred to permanent fund | 115.00 | |
| | | $870.48 |
| Balance on hand, June 9, 1903 | | $450.70 |

*Permanent Fund.*

| | |
|---|---|
| Amount of fund, June 17, 1902 | $1,710.45 |
| Transferred from current fund | 115.00 |
| Interest, Western Savings Bank | 60.89 |
| Amount of fund, June 9, 1903 | $1,886.34 |

Increase of permanent fund in past year, 10 per cent.
Increase of permanent fund, 1901-02, 22 per cent.
Increase of permanent fund, 1900-01, 162 per cent.

| | |
|---|---|
| Total receipts from all sources during past year | $994.70 |
| Total expenses | 755.48 |
| Net increase of assets | $239.22 |

Respectfully submitted,

CHARLES S. JEWETT, *Treasurer.*

June 9, 1903.

REPORT OF THE TRUSTEES OF THE BUFFALO ACADEMY OF MEDICINE FOR THE OFFICIAL YEAR *1902-3.*

*To the Fellows of the Buffalo Academy of Medicine:*

At the first meeting of the council for the present official term your trustees were charged with the care of the movable property of the Academy for the year 1902-3. Diligent search and inquiry failed to reveal the existence of a list or invoice of these articles. The last annual report of the trustees showed that the property was located in three buildings, viz.: Grosvenor Library, Public Library and new Post Office. The articles were inspected and were found in excellent condition, with the exception of three armchairs, which were broken; two of the broken chairs were repaired at a cost of one dollar; the third chair was so badly broken that it was not regarded as worth the cost of repairing.

The property in the Grosvenor Library consists of two bookcases, one of which is empty and the other contains the library, bequeathed to the Academy by the late Dr. Frederic C. Bartlett. The library consists of 186 bound volumes and 1,063 unbound periodicals, pamphlets, etc. Several of the bound volumes have passed the century mark in age. The bookcases are in good condition and furnished with locks; the keys are in the hands of the chairman of your board of trustees. At the present time this library does not subserve any utility. The superintendent of the Grosvenor Library has stated that the trustees of that institution are willing to accept the Bartlett library as a gift, and incorporate it with its medical department. It is questionable whether the Academy could rightly and legally make this disposition of it. The bookcases can remain in the basement of the Grosvenor Library building, until the space which they occupy is wanted for some other purpose.

Your trustees have made a catalogue of the library and an invoice of the movable property, which accompany this report.

All the buildings in which the movable property of the Academy is stored are fire-proof. All the movable property belonging to the Academy has lately undergone inspection and has been found in good condition. Each article is stamped with the name of the Academy.

The care of the movable property of the Academy is the only work which has devolved upon your trustees during the year.

Respectfully submitted,

J. W. Grosvenor,
M. D. Mann,
Eugene A. Smith,
*Trustees.*

[The reports of the secretaries of sections was then presented, but the meetings having been listed in the Journal each month their republication is omitted.]

The Council recommended Dr. A. G. Sage, of 12 Parker Avenue, for resident membership, whereupon he was balloted for and elected to fellowship.

On motion a vote of thanks was tendered to Dr. Jewett for the able and efficient manner in which he managed the financial business of the Academy.

It was moved by Dr. Mann, and seconded by Dr. Benedict, that the books of the Buffalo Pathological Society, now in possession of Dr. H. U. Williams, be turned over to the trustees of the

Academy, with instructions that they deposit them with the Buffalo Historical Society. Carried.

The retiring president, Dr. HERMAN E. HAYD, made a few appropriate remarks about the work of the Academy during the past year and its future needs. He then read his annual address, Fibroid tumors of the uterus.

The auditing committee reported that it had compared the report of the Treasurer with the books and vouchers and found the financial standing to be correct.

The tellers, Drs. Allen A. Jones and F. E. L. Brecht, reported the result of election as follows: president, Joseph W. Grosvenor; secretary, Edwin A. Bowerman; treasurer, Charles Sherman Jewett; trustee for three years, Ludwig Schroeter.

The newly elected officers were duly installed. Each made a few appropriate remarks, thanking the members for the honor extended to them, and pledging themselves to do conscientious work for the Academy. It was moved and seconded that a vote of thanks be tendered to the president, Dr. HERMAN E. HAYD, for his masterly address and his faithful work during the past year. Carried.

On motion a vote of thanks was extended to the secretary, Dr. F. W. McGuire, for his faithful work during his term of office. On motion a vote of thanks was extended to various retiring officers of the Academy, including chairmen and secretaries of the different sections. Carried.

Attendence, 36. Adjourned at 10.15 p.m.

